# Establishment and Application of Ligation Reaction-Based Method for Quantifying MicroR-156b

**DOI:** 10.3389/fpls.2021.794752

**Published:** 2021-12-14

**Authors:** Yuxuan He, Likun Long, Wei Yan, Liming Dong, Wei Xia, Congcong Li, Feiwu Li

**Affiliations:** Institute of Agricultural Quality Standard and Testing Technology, Jilin Academy of Agricultural Sciences, Changchun, China

**Keywords:** MiRNAs, ribonucleotide-modified DNA probe, ligation reaction, quantitative method, MiR156b

## Abstract

Microribonucleic acids (miRNAs) play significant roles in the regulation of biological processes and in responses to biotic or abiotic environmental stresses. Therefore, it is necessary to quantitatively detect miRNAs to understand these complicated biological regulation mechanisms. This study established an ultrasensitive and highly specific method for the quantitative detection of miRNAs using simple operations on the ground of the ligation reaction of ribonucleotide-modified deoxyribonucleic acid (DNA) probes. This method avoids the complex design of conventional reverse transcription. In the developed assay, the target miRNA miR156b was able to directly hybridize the two ribonucleotide-modified DNA probes, and amplification with universal primers was achieved following the ligation reaction. As a result, the target miRNA could be sensitively measured even at a detection limit as low as 0.0001 amol, and differences of only a single base could be detected between miR156 family members. Moreover, the proposed quantitative method demonstrated satisfactory results for overexpression-based genetically modified (GM) soybean. Ligation-based quantitative polymerase chain reaction (PCR) therefore has potential in investigating the biological functions of miRNAs, as well as in supervising activities regarding GM products or organisms.

## Introduction

Micro ribonucleic acids (miRNAs) are a group of endogenous, non-coding, single-stranded small molecular RNA (18–25 nt in length) that participate in the post-transcriptional regulation of gene expression. miRNAs are emerging as novel biomarkers, with profound implications for research into the responses to abiotic (Aslam et al., 2020; Singroha et al., 2021) or biotic stress (Chopperla et al., 2020; Šečić et al., 2021), development and differentiation in cellular behavior (Krol et al., 2010; Miao et al., 2020), and multiple plant signaling pathways (Curaba et al., 2014). Since their first discovery, several miRNAs have been identified in a wide range of species. Following the identification of the functions of miRNAs in crops, a large number of transgenic crops related with miRNA have been developed and commercialized worldwide (Agrawal et al., 2015; Bally et al., 2020). Among the miRNAs in plant, microRNA156 (miR156) and its target mRNAs has been widely observed as a major regulators for crop development (Cao et al., 2015) and abiotic stresses (Visentin et al., 2020). The miR156 expresses at the highest accumulation at seedling stage (Miao et al., 2019). Moreover, the expression of the other miRNAs, such as miR159, miR33 might have a effect on miR156 level (Guo et al., 2017). In the whole growth stage of grapevine, miR156b/c/d exhibited typical temporal spatial-specific expression levels (Wang et al., 2016). The Arabidopsis AtmiR156b gene overexpressed in GM Brassica napus resulted in seed lutein and beta-carotene accumulation, these achievement suggest that miR156b have a potential applification in plant breeding for enhancing carotenoid production (Wei et al., 2010). To further explore the physiological functions of miRNA or detect the content in crop genomes, specific quantitative detection methods for miRNAs that have high sensitivity, efficiency, and reliability are urgently needed for miRNA mechanism applications (Papadopoulou et al., 2020). In early miRNA studies, northern blotting (De la Rosa and Reyes, 2019) and microarray (Li and Ruan, 2009) assays were conventionally used as “gold standard” methods to identify miRNA expression. However, these complex and relatively insensitive methods cannot overcome the complications associated with the unique characteristics of miRNAs such as their low abundances in tissue, short lengths, and the high sequence similarities among miRNA families. To resolve these problems, a series of cooperative amplification programs have been reported for miRNA detection. Locked nucleic acid (LNA)–modified probes with bicyclic high-affinity RNA analogs, which are rigid structures formed by linking the 2′ and 4′ carbons on the ribose, greatly improve the hybridization efficiency of northern blotting and are sensitive to miRNA determination (Shu et al., 2019). The Agilent Microarray SurePrint technology is advantageous owing to its low RNA input requirements (∼100 ng) and eliminates bias by omitting the fractionation or amplification steps (Dell’Aversana et al., 2017). However, many solid phase hybridization assays require sophisticated fabrication or expensive capture probe modifications (Zou et al., 2017). These issues increase their costs and restrict their extensive application. Modified miRNA detection methods based on nucleic acid amplification are undoubtedly the most attractive and prominent tools for miRNA analysis and include polymerase chain reaction (PCR), ligase chain reaction (LCR) (Zhang et al., 2013), rolling circle amplification (RCA) (Cheng et al., 2009; Liu et al., 2020), and isothermal exponential amplification reaction (IEXPAR) (Jia et al., 2010). For instance, with the design of a new hairpin/deoxyribonucleic acid (DNA) ring ternary probe in RCA, the target miRNA can bind the probes through toehold-mediated strand displacement to form ternary structures and generate many repeated metal ion-dependent deoxyribozyme (DNAzyme) sequences. Finally, fluorescently quenched hairpin signal probes can be cyclically cleaved by the DNAzyme sequences, which drastically enhances the fluorescence recovery of the target miRNA (Liu et al., 2019). Tian et al. (2019) rationally combined RCA with efficient loop-mediated isothermal amplification (LAMP), which directly templates the ligation of a padlock probe to trigger the RCA reaction. Thus, they significantly improved the amplification efficiency and sensitivity owing to the double stem-loop DNAs with functional sequences that were generated by RCA-produced DNA templates.

**FIGURE 1:**
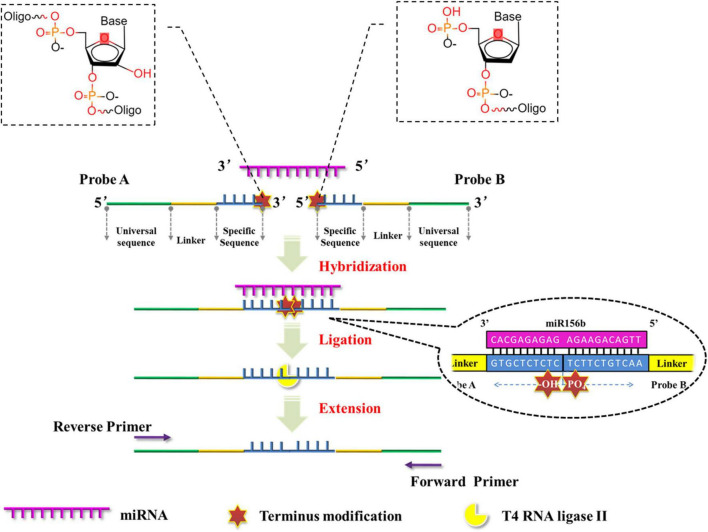
Schematic representation of miRNA quantitative polymerase chain reaction (PCR) based on the ligation reactions of ribonucleotide-modified deoxyribonucleic acid (DNA) probes.

This study proposes the development of a facile and ultrasensitive method for miRNA detection by elegantly integrating sensitivity and specificity. Here, a miRNA quantification assay is achieved through real-time PCR integrated ligation reactions. As outlined in the schematic representation in Figure 1. After determining the target miRNA to be detected, the two DNA probes in this assay contain a specific sequence (blue), a stuffer sequence (yellow) in between and a universal sequence designed as amplification primer (green). Both of the specific sequences are complementary to the half-sequence of target miRNA at the 3′- and 5′-termini, respectively. Then, the 3′-termini of probe A is modified by two nucleotides and the 5′-termini of probe B is modified by a phosphate group. Therefore, the modified groups are immediately adjacent to each other following the hybridization of the probes with the half-sequence of target miRNA. As T4 RNA ligases specifically catalyze the formation of a 3′–5′ -phosphodiester bond between a 3′-hydroxyl group and a 5′-phosphoryl group in three nucleotidyl transfer steps (Zhuang et al., 2012), and T4 RNA ligase II exhibits significantly higher ligating activity and specificity for double stranded RNA than T4 RNA ligase I (Yin et al., 2003; Cheng et al., 2009). T4 RNA ligase II is employed to link the 3′-hydroxyl and 5′-phosphoryl groups in adjacent probes and thus form ligation production, which is used as an initial template for the next real-time PCR amplification with the universal sequences. The PCR products with fluorescence or SYBR Green I signal can then be detected directly. The detection limit of the proposed method was determined and its specificity was investigated with respect to its ability to distinguish miRNAs from their corresponding pre-miRNAs, and from homogeneous miRNAs in the same family. The robustness and feasibility of the method were investigated as a tool for genetically modified (GM) content analysis.

## Materials and Methods

### Plant Materials and Extract Preparation

All plant materials were kindly provided by Jilin Academy of Agricultural Sciences, Changchun, China, and germinated in soil (pH 7.19; soil: water 1: 4) under a photoperiod of 16 h of daylight at 23°C. A 181-bp stem-loop fragment of the *GmmiR156b* (GenBank No. NR048600.1) precursor was amplified using DNA samples obtained from the soybean cultivar Williams 82 and subcloned into the *pTF101.1-35S* vector, which carried the selection marker *Bar* gene and the cauliflower mosaic virus 35S promoter that could enhance the expression of miR156b. The recombinant construct *pTF101.1-*miR156b was then integrated into *Agrobacterium tumefaciens* strain EHA101 for cotyledon-node transformation (Flores et al., 2008). After the regenerated plants were transplanted into a greenhouse, glufosinate (150 mg/L) screening and LibertyLink strip (EnviroLogix Inc., Portland, ME, United States) detection were used to identify transgenic plants. Herbicide-resistant T4 generations containing a single copy number of the target sequence were employed as miR156b GM soybean event for further experimental analyses. In this GM soybean, the expression level of miR156b has been considered by the conventional stem-loop qPCR, which further confirmed the reliability of the plant materials. Total RNA and DNA samples were extracted from eight-week-old transgenic and wild-type (WT) soybean seedlings. All procedures were performed in accordance with the EasyPure Plant RNA kit (Transgen Biotech, Beijing, China) and Dneasy Plant Mini Kit (Qiagen, Hilden, Germany). The extracted concentration and quality were calculated using absorbance measurements with an ND-1000 spectrophotometer (Thermo Fisher Scientific Inc., Wilmington, DE, United States) at wavelengths of 260 and 280 nm. All the extracted samples were then stored at −80°C for further research.

### Design of Probes and Primers for miR156 RNA Detection

All the miRNA sequences in the plant can be obtained from the online database PmiREN.^1^ The miR156 family comprises of typical miRNAs that are considered to have an explicit function applied in some transgenic soybeans; in this assay, the miR156b gene from the miR156 family was used as the model target. The structures of probes A and B are elucidated schematically in Figure 1. The specific sequences (blue) of probes A and B were designed to be complementary to half of the target miRNA sequences at the 3′- and 5′-terminals, respectively. A stuffer sequence (yellow) in between links up the specific sequence with the universal sequence (green). Probe A was modified with two nucleotides at its 3′-terminus, while probe B was modified with a phosphate group at its 5′-terminus. As the chemical features of the modified nucleotides, 3′-hydroxyl in probe A and 5′-phosphoryl groups in probe B (Figure 1) are immediately adjacent to each other following the DNA probes hybridize with the target miRNA, and the modified structure facilitates the formation of a 3′- to 5′-phosphodiester bond catalyzed by T4 RNA ligase II. Moreover, probe A-N has the same sequences as probe A except for the presence of 3′-hydroxyl groups in the two 3′-terminal ribonucleotides. Probe A-N was employed as an experimental control to verify the role of terminus modifications in the ligation reaction. In order to test the specificity of this proposed method in miRNA discrimination, for another miR156 member (miR156g), probes A-g and B-g were also designed in the same way following the replacement of specific sequences (Table 1).

**TABLE 1:** The sequences of the primers and probes in the ligation based quantitative polymerase chain reaction (PCR) amplification.

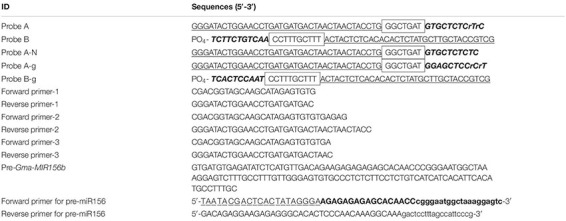

*The sequences indicated with bold italics were the complementary sequence to the target miRNA.*

*The bold in forward primer for pre-miR156 was the specific sequence of pre-miRNA.*

*The box was the stuffer sequence.*

The forward and reverse primers for PCR amplification were designed according to the universal sequences in probes B and A, respectively. For the optimization of the ligation-based qPCR, primer pairs with different lengths were designed and orthogonally combined to screen the optimal primers for the amplification of the ligation product. The sequences of the primers and probes are shown in Table 1. All high-performance liquid chromatography–purified miRNAs, polyacrylamide gel electrophoresis–purified ribonucleotide-modified DNA probes, and primers were synthesized by Shanghai Sangon Biological Engineering Technology (Shanghai, China).

### Experimental Procedure of Ligation-Based Quantitative Polymerase Chain Reaction Assay

The mixture contained 2 nM each of template miRNA and probes A and B. The procedure was initially performed at 65°C for 8 min. In this step, both the DNA probes identify and hybridize with the target miRNA. Then, 2 U of T4 RNA ligase II and 10× ligation buffer (New England Biolabs, Shanghai, China) were added to the hybridization product, and a total final volume of 50 μl was incubated at 37°C for 1 h to perform the ligation reaction; the products were then immediately placed on ice until it cooled to room temperature (∼15 min).

The FastStart Universal SYBR^®^ Green Master (Roche, Mannheim, Germany) was used for real-time quantitative PCR assays. The quantitative PCR assays for amplifying the target miRNA were performed with a 20-μl final volume containing 2× SYBR Green Mastermix (ROX), 0.2-μM forward and reverse primers, 5 μl of ligation production, and 4.2 μl of diethylpyrocarbonate-treated water (DEPC-H_2_O; Takara Biotechnology, Changchun, China). The reactions were incubated at 95°C for 5 min on a Bio-Rad CFX96 Real-time Thermal Cycler (Bio-Rad, Hercules, CA, United States), followed by 40 cycles of 95°C for 10 s, 60°C for 30 s, and then 72°C for 30 s. All reactions were performed in triplicate.

### Preparation of Pre-miR156b *in vitro* Transcription Assays

The precursor of miR156b, pre-miR156b, was prepared using an *in vitro* transcription reaction. First, the forward and reverse primers for pre-miR156b (pre-FP and pre-RP) were designed based on the sequences of pre-GmmiR156b,^2^ in which the 20 bases located at the 3′-terminus of pre-FP complemented each other with the 20 bases located at the 3′-terminus of pre-RP. A mixture containing 50 pmol of primer and 10 μl of Klenow buffer (Fermentas, Shanghai, China) was incubated at 75°C for 5 min and cooled on ice. After the 20 complementary bases hybridized completely with each other, deoxynucleotide triphosphate (dNTP; 250 μM final concentration; Takara Biotechnology, Changchun, China), Klenow buffer, and 5 U of Klenow DNA polymerase (exo-; Fermentas, Shanghai, China) were added and replenished with DEPC-H_2_O (Takara Biotechnology, Changchun, China) to a final volume of 20 μl. Following incubation at 37°C for 1 h, the 3′-terminus sequences of both pre-FP and pre-RP generated double-stranded DNA (dsDNA) by extension reactions. Then, the reaction mixture was heated at 75°C for 20 min to inactivate the Klenow DNA polymerase. The complete sequence consists of the T7 promoter, GGGA (guanine–guanine–guanine–adenine) spacer, and a pre-miR156b specific sequence from the 5′- to 3′-terminus sequence of this dsDNA. The *in vitro* transcription reaction for pre-miR156b was performed in a final volume of 50 μl containing 20 μl of dsDNA, 30 μl of *in vitro* transcription buffer, 100 U of ribonuclease inhibitor (Takara Biotechnology, Changchun, China), and 80 U of T7 RNA polymerase (Fermentas, Shanghai, China). The reaction mixture was incubated at 37°C for 4 h to amplify pre-miR156b, and then digested with 5 U of RNase-Free DNase I (Takara Biotechnology, Changchun, China). Finally, the above components were purified with an RNA cleanup and concentration kit (Shenggong, Shanghai, China) for 5 min. The concentration of the final pre-miR156b product was determined by analyzing its absorption at 260 nm with an ND-1000 spectrophotometer (Thermo Fisher Scientific Inc., Wilmington, DE, United States).

### Data Processing Methodology

For data analysis, a 10-fold dilution series of ligation products was used as a template for quantitative PCR assays. This was done to generate a plot of log copy numbers of miRNAs vs. the corresponding threshold of cycle (C_*T*_). The concentration of the target miRNA was detected through its linearity with C_*T*_, which spanned at least seven orders of magnitude. Slope and PCR amplification efficiency (*E*) were calculated according to the following equation: slope = −(1/log *E*). The relative detection rate of other miR156 family members was calculated as 1/2^ΔCT^, in which ΔC_*T*_ = C_*T, other* miRNA_−C_*T*,__*miR*156*b*_. The relative detection rate of miR156b was defined as 100% (Zhang et al., 2011).

## Results and Discussion

### Analytical Performance of Deoxyribonucleic Acid Probes Modified With Two Ribonucleotides for Reaction Efficiency

**FIGURE 2:**
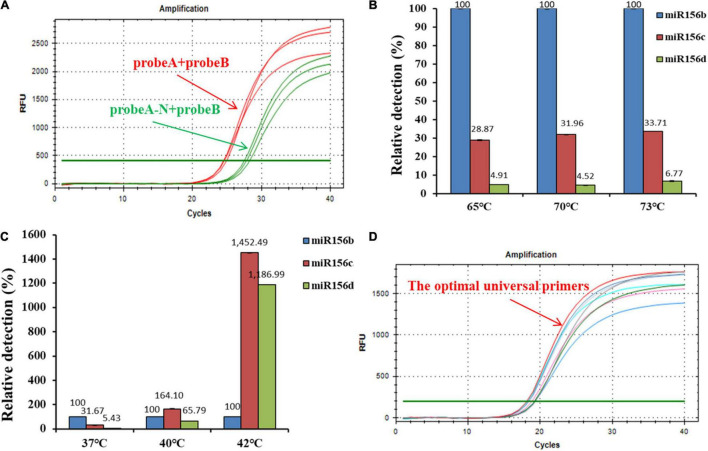
Optimization of ligation-based assays. **(A)** Analytical performance of deoxyribonucleic acid (DNA) probes modified with two ribonucleotides for reaction efficiency; probe A-N was a control probe without any ribonucleotide modification at its 3′-termini. **(B)** Effect of hybridization temperature on detection specificity. **(C)** Effect of ligation temperature on detection specificity. **(D)** Screening of optimal universal primers for amplification assays; red line: universal primer with the lowest GC content.

The high specificity of T4 RNA ligase II is dependent on the 3′-hydroxyl and 5′-phosphoryl groups that are adjacent to either a duplex RNA or an RNA–DNA hybrid. Thus, the effect of the modification of two nucleotides on reaction efficiency was examined in the miR156b detection assay. After either probe A/probe B or probe A-N/probe B was hybridized with the target miRNA and ligated by the T4 RNA ligase II, the ligation product was used as an initial template for PCR amplification. As seen in the real-time fluorescence signal in Figure 2A, templates with concentrations as low as 2 nM could be accurately detected by the probe A/probe B set in the ligation step, with C_*T*_ values ranging from 24.47 to 24.91 in triplicate. However, under the same experimental conditions, the ligation product using probe A-N/probe B had a C_*T*_ value ranging from 27.48 to 28.25 after PCR amplification was completed. These results demonstrate that although T4 RNA ligase II could directly catalyze DNA nick ligation in a way, ribonucleotide modification at the 3′-terminus greatly improved catalytic efficiency, and was more conducive to ligation-based PCR amplification.

### Optimization of Ligation Reaction of Ribonucleotide-Modified Deoxyribonucleic Acid Probes

The ability to distinguish similar miRNAs is essential for miRNA detection. The central goal of the developed ligation-based qPCR assay was to establish the appropriate reaction conditions to improve the specificity and reduce the unexpected ligation and hybridization with non-target miRNAs. To ensure high ligation reaction efficiency, the hybridization and ligation temperatures were optimized. In this ligation reaction, miR156b, miR156c, and miR156d were selected as experimental templates based on the similarity of the sequences. The results of the real-time fluorescence signal (Figures 2B,C) show that the hybridization temperature (from 65 to 73°C) had no consequence on the specificity to identify target miR156b, whereas the specificity of the ligation reaction decreased with increasing ligation temperature (from 37 to 42°C). When the ligation temperature was >40°C, the fluorescence signals of interference generated by miR156c were significantly enhanced compared to those of miR156d and miR156b. When the ligation temperature was set at 40 or 42°C, with equivalent molar amounts of the miRNA template, the cycle value of miR156b amplification was later than that at 37°C (Supplementary Figure 1). Data indicate that the initial amount of miR156 used for amplification was reduced in the real-time PCR assay. Based on the above mentioned comprehensive experimental results, 37°C was considered as the optimal ligation temperature to ensure detection specificity.

The amplification efficiency of quantitative real-time PCR depends on the oligonucleotide primer sequences. In order to produce well-defined real-time fluorescence signals, several combinations of forward and reverse primers with different lengths were designed for PCR amplification. As depicted in Figure 2D and Supplementary Table 1, real-time fluorescence curves were generated by the three primer pair combinations. The C_*T*_ values of target miR156b ranged from 18.15 to 19.33. Although there was no significant difference between the experimental data of each group, the primer pair with the smallest C_*T*_ values was still employed as the optimal universal primer pair (forward primer: 5′-CGACGGTAGCAAGCATAGAGTGTGTGA-3′; reverse primer: 5′-GGGATACTGGAACCTGATGATGACTAACTAACTACC-3′) for subsequent experiments. As the GC content was closely related to the annealing temperature, it is speculated that the lower GC content can promote the binding of primer and target template to a certain extent.

### Dynamic Range and Sensitivity

**FIGURE 3:**
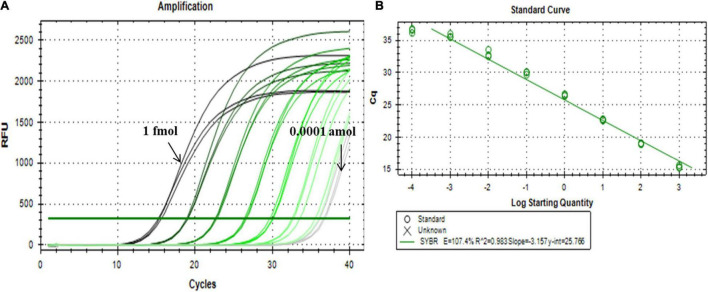
Dynamic range and sensitivity of the synthetic miR156b assay over eight orders of magnitude. **(A)** From left to right, the concentrations of synthetic miR156b is 1 fmol, 100 amol, 10 amol, 1 amol, 0.1 amol, 0.01 amol, 0.001 amol, and 0.0001 amol. **(B)** Dynamic relationship between C_*T*_ value and log of miR156b concentration; *E*: polymerase chain reaction (PCR) efficiency, *R*^2^: squared regression coefficient of the standard curve.

Using the systematically optimized reaction parameters, a standard curve for evaluating the dynamic range and sensitivity of the ligation-based qPCR method was established *via* the 10-fold series dilution of ligation products as quantitative templates, spanning over at least seven orders of magnitude (miR156b inputs in the range of 1 fmol–0.0001 amol). This calibration method is superior to the delta threshold of cycle (C_*T*_) method in terms of its detection limits (Taverniers et al., 2004). The quantitative PCR assay targeting miR156b further demonstrated an excellent linearity between the C_*T*_ value and the log of the target concentration in the range of 1 fmol–0.0001 amol. The correlation equation was C_*T*_ = −3.157 log C_*miRNA*_ + 25.766. The proposed ligation-based quantitative system showed a corresponding PCR efficiency of 107.4%, and the squared regression coefficient (*R*^2^) of the standard curve was 0.983 for the three replicates (Figure 3). These results show that there was a good linear relationship between the target substance and PCR C_*T*_ value, over a wide range of concentrations. Notably, miR156b could be detected at concentrations as low as 0.0001 amol, demonstrating that the developed quantitative method has good sensitivity.

### Specificity Study

**FIGURE 4:**
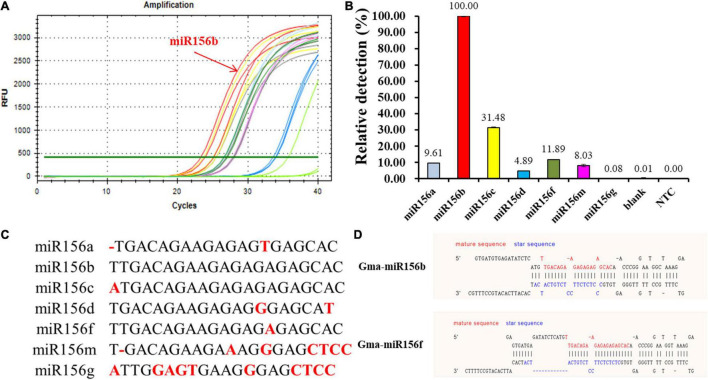
Evaluation of specificity among miR156b family. **(A)** Amplification plot of synthetic miR156 family members; red line: miR156b; the other color lines: the highly homologous miR156 members. **(B)** Relative detection rate of miR156 family members. Blank was a negative control used miRNA-free ligation product as the amplification template; NTC was a quantitative reaction without any ligation product. **(C)** The alignment of all miR156 sequences. Nucleotides that differ from those in miR156b are highlighted. **(D)** The structural difference between GmmiR156b and GmmiR156f.

The miR156 family contains seven highly homologous miRNAs with only one to a few mismatched nucleotides. To evaluate the specificity of the proposed qPCR method for miRNA detection, probes A and B were selected to verify whether the said method could identify other miR156 family members with different sequences. As shown in Figure 4, miR156b was identified, and each miR156 sequence obtained a different relative detection rate. Compared to miR156b, both miR156c and miR156f differed by only a single adenine nucleotide; the single-base nucleotide in miR156c was located at the 3′-terminus of probe B. Non-specific detection (31.48%) was higher than that of miR156f (11.89%), whose mature sequence was consistent with miR156b except for the star sequence (Figures 4C,D). This difference may further affect the secondary structure. The analytical data stored in the online database PmiREN (see text footnote 1) also confirms this aspect of miRNA. These results demonstrate that the secondary structure can affect specificity by influencing the binding between the probe and target miRNA to some extent. Both miR156a and miR156d had a mismatched nucleotide at the same position on the 3′-terminus of probe A, but another differential nucleotide in miR156a was located at the 5′-terminus of the mature miRNA sequence, while that of miR156d was at the 3′-terminus of the mature miRNA sequence. The higher non-specific detection determined for miR156a (9.61%) indicates that the method’s specificity was affected not only by the number of differential nucleotides, but also by the location. In addition, two negative controls were established to verify the effect of the ligation between DNA probes on the fluorescence signals, one was a quantitative reaction without any ligation product (NTC), the other was a negative control used for the miRNA-free ligation product as the amplification template (blank). Neither of the negative controls generated notable fluorescence signals. Therefore, it can be inferred that the two DNA probes ligate each other in the absence of the target miRNA to produce a minimal effect on the quantitative results. Collectively, the proposed ligation-based qPCR method can characterize with a high specificity, discriminate differences as small as a single nucleotide, and detect secondary structural differences among miRNA targets.

### Practicability Validation for miRNA Detection

**FIGURE 5:**
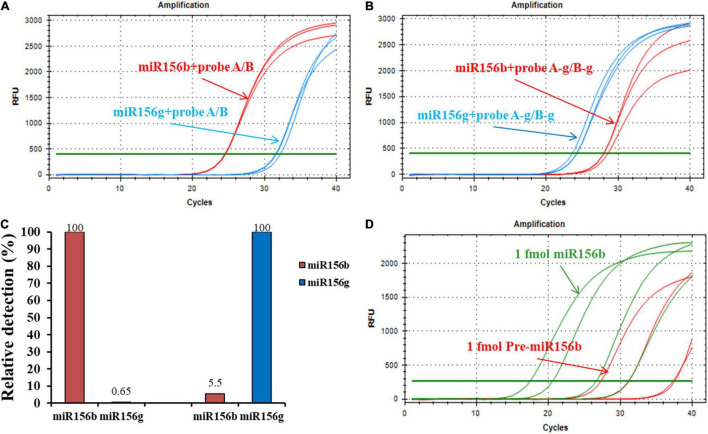
Generality and practicability of the ligation-based assays for detection of different miRNAs. **(A)** Amplification plot of miR156b and miR156g with probe A/B. **(B)** Amplification plot of miR156b and miR156g with probe A-g/B-g. **(C)** Relative detections for miR156b and miR156g with different target miRNAs. **(D)** Specificity of developed method in distinguishing miRNAs from their corresponding pre-miRNAs. From left to right in red line, the concentrations of synthetic miR156b is 1 fmol, 100 amol, 10 amol, 1 amol; from left to right in green line, the concentrations of synthetic pre-miR156b is 1 fmol, 100 amol, 10 amol, 1 amol.

A different miRNA (miR156g) was randomly selected to verify the universal practicability of the developed ligation-based quantitative PCR methods. To this end, specific probes A-g and B-g were designed with modified ribonucleotides containing the target-specific sequence that is complementary to the half sequence of miR156g. The stuffer sequence and universal sequences for PCR amplification were the same as those for probes A and B. Distinct results can be observed in Figures 5A–C, where equivalent amounts of miR156b and miR156g with different probes hybridized with respective or adverse probes produced well-defined signals. When using miR156b-specific probes (probe A and probe B) to perform the ligation reaction, and calculate the relative detection level with the formula:

ΔC=TC-T,miR156gCbT,miR156

The non-specific detection rate using miR156g reached 0.64%. For the target miRNA, the non-specific detection using miR156b was 5.5% while using miR156g-specific probes. These results demonstrate that the method has good generality and practicability for the detection of different miRNAs. The high specificity of ligation-based method depends on the complementary degree of the probes and the target sequence, but not the target miRNA sequence itself.

Pre-miRNA is the precursor of mature miRNA; it has stem-loop hairpin secondary structures that can be further cleaved by the dicer-like 1 (DCL1) and HYPONASTIC LEAVES1 (HYL1) into an miRNA: miRNA* (* represents secondary structure) duplex in the nucleus (Sun, 2012). To further investigate the pre-miRNA that were present in the sample on their specificity for miRNA detection, the pre-miRNA of miR156b (pre-miR156b) was prepared using the *in vitro* transcription reaction. The template concentration for the ligation reaction for pre-miR156b was determined to be in the range of 1 fmol–1 amol. The amplification plots of miR156b and pre-miR156b with the ligation-based qPCR assay showed that pre-miRNA produced negligible signals at concentrations below 10 amol (Figure 5D). In the template concentration range from 1 fmol to 10 amol, each C_*T*_ value of pre-miR156b was nearly ten cycles later than the corresponding miR156b, equating to 0.1% of the non-specific detection. These experimental data indicate that the developed ligation-based quantitative method has high specificity in distinguishing miRNAs from their corresponding pre-miRNAs.

### Quantification of miR156b in Genetically Modified Soybean

**FIGURE 6:**
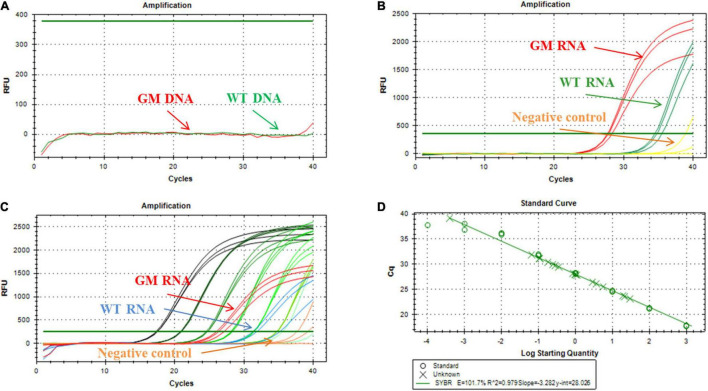
Application of ligation-based assay for miRNA detection in genetically modified (GM) soybean. **(A)** Amplification plot of miR156b using deoxyribonucleic acid (DNA) as template, the total DNA was extracted from WT and GM soybean, respectively. **(B)** Amplification plot of miR156b using RNA as template, the total RNA was extracted from WT and GM soybean, respectively; red line: GM plants, green line: WT plants, yellow line: negative control (NTC). **(C)** Dynamic range and quantification of miR156b assay; red line: total RNA samples of GM plants, blue line: total RNA samples of WT plants, yellow line: negative control. **(D)** Correlation of total RNA input to C_*T*_ values for miR156b detection. The total RNA input ranged from 10 to 0.4 μg per ligation reaction.

As shown in Figure 6A, when soybean DNA was used as a template, no signal was produced in the reaction. This confirmed that the RNA sample reaction was not affected, even if some DNA was present. An equivalent amount of RNA extracted from GM and WT seedlings generated well-defined fluorescence signals in the ligation-based qPCR (Figure 6B), but it was evident that miR156b was expressed in GM soybean much more than in WT soybean. The synthesis miRNA156b range from 1 fmol to 0.0001 amol was selected for standard curve construction. As shown in Figures 6C,D, there was good linearity between C_*T*_ and the log of total RNA from 10 to 0.4 μg in the quantified assays. The data show that PCR efficiency was 101.7% and the *R*^2^ of the standard curve was 0.979. Based on this standard curve, miR156b levels were determined in both the GM and WT samples, revealing that the fold change of miR156b expression level in the GM plants was 5.08 times higher using the established ligation-based quantitative method. These results are quite similar to the that of the developer’s research data (4.82-fold change). Therefore, the established ligation-based method has the potential to detect and quantify GM samples using a miRNA mechanism.

## Conclusion

In summary, this study experimentally demonstrated an ultrasensitive fluorescence method for miRNA quantification by elegantly integrating sensitivity and specificity. Under the hybridization procedure followed by DNA nick ligation catalyzed by T4 RNA ligase II, the initial template for miRNA amplification was obtained and quantified by quantitative real-time PCR. After optimizing the influential conditions, the proposed method could accurately discriminate among various miRNAs even when the difference was as small as a single nucleotide mismatch or the secondary structure difference leading by the nucleotides location site. High specificity was also achieved in the detection of mature miRNAs against their precursor sequences. In addition, the practicability of the proposed ligation-based quantitative PCR was further confirmed in that the total DNA and RNA samples from GM and WT soybeans generated well-defined fluorescence signals in a satisfactory dynamic range. The fold change of miR156b expression level compared well with data obtained by the conventional stem-loop qPCR, which further confirmed the reliability of the method. In contrast to the advantages and disadvantages of previously described methods for miRNA detection with data side-by-side (Table 2), the DNA probes and universal primers used in this method can be simply designed, without the need for complex structures such as the stem-loop reaction (Mei et al., 2012) or RCA assay (Liu et al., 2019), operators only need to substitute the specific sequences complementary to half of the target miRNA. Moreover, this method uses SYBR Green I and a small amount of ribonucleotide-modified DNA probes, meaning that it is also more cost efficient than that of the TaqMan probe. This characteristic greatly improves the method’s efficiency and convenience in the quantification and detection of various miRNAs. Combining the detection limit and specificity results investigated with its ability to distinguish miRNA from the other similar sequences, the simple operation and feasibility of this ligation-based PCR method implies that it can become a potential tool in GM content analysis.

**TABLE 2:** The comparison among ligation-based polymerase chain reaction (PCR) and the other similar methods for miRNA detection.

Methods	Detection limit	Analysis time	Advantages	Disadvantages	References
Ligation-based PCR	0.1 zmol	2.2 h	Simple probe design and easy to operate, good sensitivity and specificity	Special enzymes are needed, the result is affected by ligation efficiency	This work
Stem-loop RT-PCR	0.2 fmol	3 h	Good sensitivity and accuracy	Time consuming, complicated probe design	Mei et al., 2012
RCA assay	1.51 fmol	>10 h	Good efficiency of signal amplification, no PCR needed	Time consuming, complicated probe design, non-specific amplification of background	Liu et al., 2019
Hybridization chain reaction	10 pmol	>1 day	Better detectability, no enzyme is required	Cost consuming, specialized equipment needed	Zou et al., 2017
Isothermal exponential amplification reaction	0.1 zmol	<1 h	Good efficiency of signal amplification, no modified DNA probes is required, good sensitivity and specificity, low-cost	Multiple enzymes, specific nicking-enzyme recognition site and more condition optimization are required.	Jia et al., 2010

## Data Availability Statement

The datasets presented in this study can be found in online repositories. The names of the repository/repositories and accession number(s) can be found in the article/Supplementary Material.

## Author Contributions

YH contributed to the conception and design. LL and WY performed the main laboratory and wrote the manuscript. LD and WX carried out the data analysis and interpretation. CL provided the direction. FL drafted the manuscript and critically revised it for important intellectual content. All authors read and approved the manuscript.

## Conflict of Interest

The authors declare that the research was conducted in the absence of any commercial or financial relationships that could be construed as a potential conflict of interest.

## Publisher’s Note

All claims expressed in this article are solely those of the authors and do not necessarily represent those of their affiliated organizations, or those of the publisher, the editors and the reviewers. Any product that may be evaluated in this article, or claim that may be made by its manufacturer, is not guaranteed or endorsed by the publisher.

## Footnotes

1
https://www.pmiren.com/search

2
https://www.pmiren.com/singlemirna?Accession=PmiREN006720
