# Supplementary figures and images for: Establishment and Application of Ligation Reaction-Based Method for Quantifying MicroR-156b
Source: Front Plant Sci. 2021 Dec 14;12:794752. doi: 10.3389/fpls.2021.794752 (PMC8713971; doi:10.3389/fpls.2021.794752)

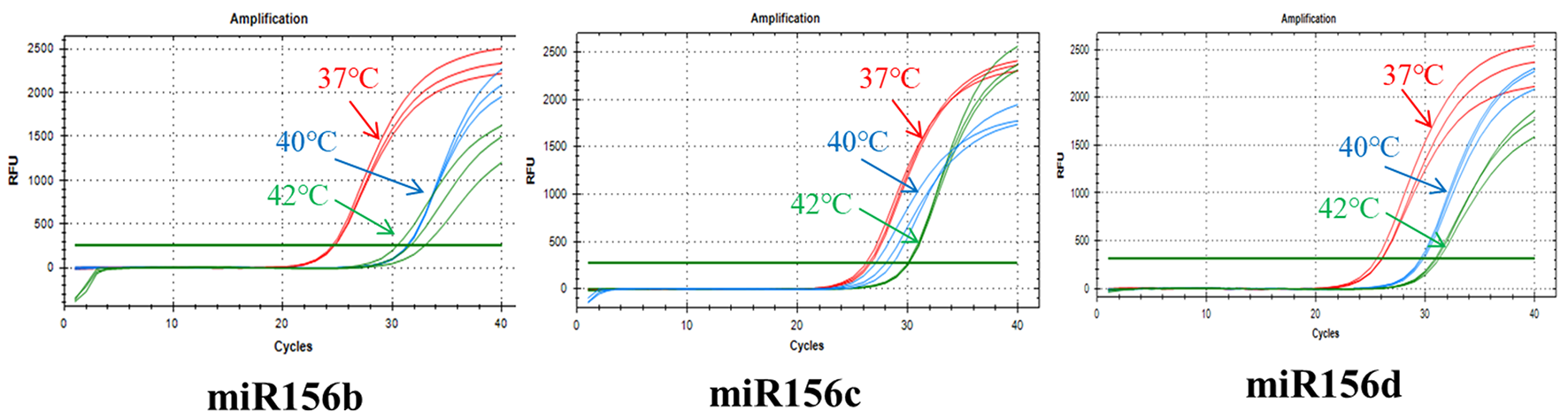

Supplement: Supplementary file 1 [file Image_1.TIF]
