# Supplementary material for: Establishment and Application of Ligation Reaction-Based Method for Quantifying MicroR-156b
Source: Front Plant Sci. 2021 Dec 14;12:794752. doi: 10.3389/fpls.2021.794752 (PMC8713971; doi:10.3389/fpls.2021.794752)
Supplement: Supplementary file 2 [file Table_1.DOCX]

**Table S1. Optimization of universal primers for quantitation PCR**

| Primer Pairs | GC conent in forward primer (%) | GC conent in reverse primer (%) | Ct value |
| --- | --- | --- | --- |
| Forward primer-1/Reverse primer-1 | 54.2 | 50 | 19.29 |
| Forward primer-2/Reverse primer-2 | 53.3 | 44.4 | 18.22 |
| Forward primer-3/Reverse primer-3 | 51.9 | 46.4 | 18.87 |
| Forward primer-1/Reverse primer-2 | 54.2 | 44.4 | 18.48 |
| Forward primer-1/Reverse primer-3 | 54.2 | 46.4 | 19.21 |
| Forward primer-2/Reverse primer-3 | 53.3 | 46.4 | 18.37 |
| **Forward primer-3/Reverse primer-2** | **51.9** | **44.4** | **18.15** |
| Forward primer-3/Reverse primer-1 | 51.9 | 50 | 19.33 |

Note: Primers with different length were designed by link stuffer sequences at 3’-terminus. The bold indicated the optimal primer pairs
